# The Treatment of Scarlet Fever

**Published:** 1894-05

**Authors:** 


### THE TREATMENT OF SCARLET FEVER.

At a meeting of the Academy of Medicine in New York, Dr. A. Jacobi read a paper, in which he discussed the question of the treatment of scarlet fever. He expressed the opinion that in a mild case of scarlatina little was necessary beyond keeping the room at a uniform temperature, but well ventilated; the patient should have a light diet, with calomel occasionally to prevent auto-intoxication; the skin should be oiled and the child kept in bed, because any case may be followed by renal disease due to exposure. Sometimes a moderately high temperature is not well tolerated at the commencement of the case; therefore some antipyretic, such as a warm bath, or moderate doses of tincture of aconite should be given, but not antipyrin, acetanilid or phenacetin. Now and then the skin should be sponged with alcohol and water to keep the temperature down. Quinine is not indicated, because it may disturb the stomach. In severe cases it is too much the custom to restrict the administration of internal remedies. The principal complication in these cases is the condition of the throat, the local treatment of which is likely to injure the epithelial exudate. Treat the throat *via* the nose by injections, rather than sprays, of boracic acid solution, or weak bi-chloride of mercury solution. Sometimes the condition goes on to the formation of large glandular abscesses or to phlegmonous inflammation: the part should be incised at once, though there may be but little pus, as much necrotic tissue as possible being removed by scraping; the cavity should be packed with salicylated gauze, because iodoform gauze may cause troublesome absorption, and carbolic acid causes coagulation. This is also to be apprehended with tincture of iron, though tincture of iodine does not coagulate blood. The incisions should not be made under an anesthetic, because the patient is usually too weak to tolerate anesthesia.

1. The author omits to state the number.

Otitis media is another complication of scarlatina that must be prevented by attention to the nose and throat. For the rheumatism that may occur, salicylate of soda should be used. Often an endocardial murmur may be heard; this is actually myocardial, as degeneration occurs in the heart tissue in consequence of the fever, no digitalis should be given, but opium and ice over the heart. For some nervous symptoms that arise, phenacetin may be given if the warm bath or warm pack prove insufficient. The post-scarlatinal chorea due to cerebral emboli or to anemia should be treated symptomatically or according to the causation. Meningitis is due primarily to the throat infection, another reason for treating that region at once. Post-scarlatinal mental disease must be treated by rest, freedom from excitement and suitable stimulation. All efforts may fail to prevent the occurrence of nephritis; when it occurs the child should be kept in bed and given a warm bath in the evening, and under no circumstances should he be allowed to leave the room until all the symptoms are over. In treating this complication, calomel is of value on account of its action on the intestinal tract as well as on the kidneys. The worst cases are those in which there is no dropsy, because there is likely to be cerebral edema. If uremic convulsions occur, chloroform is indicated; if morphine is given, it should be in conjunction with atropine or hyoscyamine. Digitalis should never be given. Sometimes pilocarpine is useful, but it must be administered with caution. For chronic nephritis there is nothing better than iodide of potassium alternating with bi-chloride of mercury. —*North American Practitioner.*

